# Open-Field Blast Injury Disrupts Corneal Gene Expression Linked to Ion Transport, Sensory Perception, and Neural Signaling

**DOI:** 10.1167/iovs.66.11.68

**Published:** 2025-08-27

**Authors:** Rajnish Kumar, Nishant R. Sinha, Suneel Gupta, Ratnakar Tripathi, Charles J. Smith, Alexandria C Hofmann, Catherine Johnson, Frederick W. Fraunfelder, Rajiv R. Mohan

**Affiliations:** 1 Harry S. Truman Memorial Veterans’ Hospital, Columbia, Missouri, United States; 2 Department of Veterinary Medicine and Surgery, College of Veterinary Medicine, University of Missouri, Columbia, Missouri, United States; 3 Mason Eye Institute, School of Medicine, University of Missouri, Columbia, Missouri, United States; 4 Department of Radiology, School of Medicine, University of Missouri, Columbia, Missouri, United States; 5 Department of Mining and Explosives Engineering, Missouri University of Science and Technology, Rolla, Missouri, United States

**Keywords:** cornea, open-field blast injury, RNA sequencing (RNA-seq), transcriptomics, differential gene expression (DGE), protein-protein interaction (PPI) networks

## Abstract

**Purpose:**

Open-field blast injury (OFBI) is a common cause of vision loss. This study investigated OFBI-induced changes in corneal transcriptome, signaling pathways, and protein-protein interactions using RNA sequencing (RNA-seq).

**Methods:**

Sixty-four C57BL/6J mice were used. OFBI was produced in live mice with 350 g of Composition C4 explosive at a 3 meters (m) standoff distance. RNA was extracted from OFBI-exposed and unexposed corneas, and RNA-seq libraries were generated and sequenced on the Illumina NovaSeq 6000 platform. Reads were aligned to the mouse reference GRCm38 genome using HISAT2. Differential gene expression (DGE) analysis was performed using DESeq2. Pathway enrichments were studied using Gene Ontology (GO), Kyoto Encyclopedia of Genes and Genomes (KEGG), and Reactome databases. Protein-protein interaction (PPI) networks were constructed using STRING database and Cytoscape coupled with the cytoHubba plugin.

**Results:**

DGE analysis identified 2860 significantly DGEs in OFBI-exposed mice from unexposed (adjusted *P* < 0.05) mice. The 30 upregulated and 735 downregulated genes showing a log2 fold change ≥ ±1.5 were identified. Enriched pathways included ion channel and membrane potential regulation, sensory perception, and neurotransmitter signaling. The *Grik2*, *Gabrg3*, *Drd2*, *Gabrr1*, *Gabra2*, and *Drd4* genes which are commonly associated with ion channel regulation, neuronal signaling, and sensory functions were significantly downregulated on day 14 post-OFBI. The three distinct PPI pathways were associated with ion channels, sensory perception, and neurotransmitter transport. RNA sequence and gene count files are published at the NCBI Gene Expression Omnibus (GSE292886).

**Conclusions:**

OFBI led to significant disruptions in corneal transcriptome critical in the modulation of ion channel activity, sensory perception, and neurotransmitter signaling.

The prevalence of corneal injuries from explosives has markedly increased globally due to conflicts in Afghanistan, Syria, Iraq, Myanmar, Nagorno-Karabakh, Russia-Ukraine, Israel-Hamas, and Isreal-Iran apart from numerous terrorist attacks around the world in last 2 decades. Accumulating literature indicates that civilian and military personnel exposed to explosive blasts in open field sustain ocular injuries, ranging from superficial corneal abrasions to severe stromal and endothelial damage causing haze/fibrosis and neovascularization in the cornea.^1^^–^^3^ The open-field blast injury (OBFI) presents complex clinical challenges due to multifactorial pathological effects on various ocular tissues,^3^^,^^4^ often resulting in short- and long-term visual loss from corneal damage resulting in haze, edema, fibrosis, and/or neovascularization reducing the patient's quality of life.^5^^–^^7^

Open-field blasts consist of shockwaves, debris, shrapnel, projectiles, etc., and are known to cause ocular injuries categorized as primary, secondary, tertiary, and quaternary based on the extent of trauma. The open-field blasts generate a rapidly expanding shockwave characterized by high-pressure air moving outward, directly impacting the cornea and causing defects, such as epithelial disruption, stromal injury, and endothelial cell loss.^8^^–^^10^ The reflective pressure, which can be significantly greater than the incident pressure, exacerbates the extent of injury. Secondary injuries occur due to shrapnel, debris, and particulate matter cause severe eye trauma.^11^^,^^12^ Blasts often cause nearby structures to shatter, including glass, which can become high-velocity projectiles. Such debris can lead to penetrating injuries to the eye, including lacerations, punctures, and blunt trauma. High-velocity flying glass is a common source of ocular injury in explosive detonations leading to both superficial and deeper tissue damage. Tertiary injuries involve displacement of the body and subsequent impact trauma. Quaternary injuries are caused by associated factors such as exposure to toxic chemicals or burns.^13^ Comprehensive research and preventive measures are essential for protecting the eye particularly the cornea that provides two-thirds refraction to the eye and is highly vulnerable to open-field blast exposure being an outermost and protective layer of the eye.

Various animal models have been developed to simulate blast injuries, although each has limitations relative to true OFBI conditions. Shock tube and blast chamber models generate controlled overpressure but lack environmental realism and therefore fail to replicate secondary/tertiary injury dynamics.^14^^–^^20^ Ballistic gelatin models focus on penetration injuries from the shrapnel but do not encompass the broader over-pressure effects found in real blasts.^21^^–^^23^ The closed-head impact, fluid percussion injury, and controlled cortical impact models are limited to simulating mechanical forces and localized injuries without addressing the systemic impact of overpressure and multifactorial trauma.^24^^,^^25^ Weight drop models provide insights into blunt trauma but fail to produce rapid propagation of blast waves.^26^^,^^27^ Blast overpressure exposure systems generate controlled shockwaves but lack the environmental complexities and debris interactions of open-field blasts.^28^^–^^30^ The penetrating ballistic-like injury models capture projectile impacts but not the holistic, systemic, and environmental effects of real blasts or explosions.^31^^–^^35^ These models have been often used to study traumatic brain injury and ocular damage to the retina. To date, no study has characterized changes in molecular responses in the cornea following real-world OFBI in vivo in animals. This study, to the best of our knowledge, for the first time, presents the changes in molecular and signaling pathways in the cornea post-OFBI in mice in vivo via RNA sequencing (RNA-seq) analysis, that offers a transcriptomic blueprint of OFBI-induced alterations under real blast conditions in an open field.

## Methods

### Open-Field Blast Exposure of Mice

All procedures were performed under approved protocol of the Institutional Animal Care and Use Committee (IACUC) and Institutional Biosafety Committee (IBC). Open-field blast injury to eyes were produced at the Experimental Mine site of the Truman VA Open-Field Blast Core (OFBC) facility at the University of Missouri Science and Technology, Rolla, Missouri, USA. Sixty-four 8 to 10 week old C57BL/6J mice (Charles River Laboratories, Inc., Wilmington, MA, USA) were used, and the study had two groups: naïve mice and OFBI mice. Each group had 32 mice separated into 8 subgroups (4 male and 4 female mice) to serve as biological replicate.

**Figure 1.**
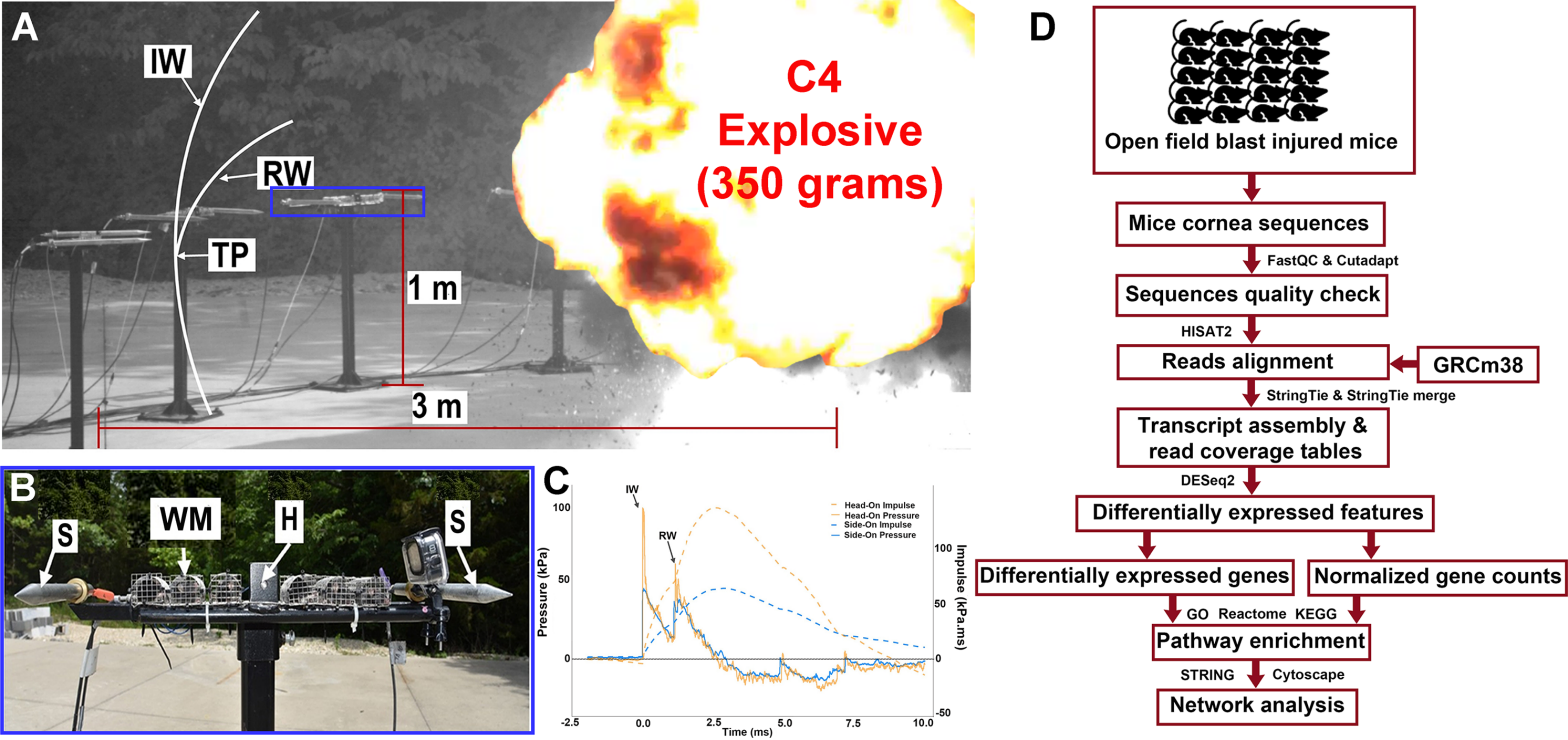
Blast exposure setup and RNA-seq analysis workflow. (**A**) High-speed video (Phantom V2015, 80,000 fps) capturing the detonation of a 350-gram C4 explosive at a 3-meter standoff distance. Subjects were placed 1 meter above the ground and 3 meters away from a 350-gram C4 explosive charge. Test setup showing the interaction of the incident wave (IW) and reflected wave (RW) forming a triple point (TP). (**B**) Close-up of the test stand (*blue box* in **A**) displaying the positioning of sensors: side-on pencil probes (S), a flush mount sensor for head-on measurement (H), and wire mesh (WM) used to hold the subjects. (**C**) Representative pressure–time and impulse–time profiles recorded from both head-on (solid lines) and side-on (*dashed lines*) orientations, showing the primary incident wave (IW) and ground-reflected wave (RW). (**D**) RNA-seq workflow: RNA from open field blast injured (OFBI) and naive mouse corneas underwent quality control (FastQC, Cutadapt), read alignment (HISAT2 to GRCm38), transcript assembly and quantification (StringTie), differential gene expression analysis (DESeq2), pathway enrichment (GO, Reactome, and KEGG), and network analysis (STRING, Cytoscape).

OFBI was performed in awake mice (without sedation) by a single blast generated via detonation of 350 grams of Composition C-4, a standard military-grade explosive, positioned at a standoff distance of 3 meters (Figs. 1A, 1B). The custom-built test platform, elevated 1 meter above ground level, was equipped with a wire mesh cage to securely hold the mice during exposure. Pressure sensors included two pencil probes mounted laterally for side-on measurements and a flush-mounted transducer for head-on measurements (Fig. 1C). The low-level blast exposure had an average side-on pressure of 47 kPa and head-on pressure of 120 kPa. Impulse calculations for side-on and head-on were 59 kPa ms and 66 kPa ms, respectively.

### RNA Extraction and Sequencing

Total RNA was isolated from mouse corneas using RNeasy Mini Kit according to the manufacturer's instructions (QIAGEN, Germantown, MD, USA) post-OFBI exposure on day 14 and age-matched naïve eyes. NanoDrop ND-1000 (Thermo Fisher Scientific, Carlsbad, CA, USA) was used to confirm high purity and measure the amount of RNA in all samples.

The RNA samples were submitted for sequencing to Arraystar, Inc. (Rockville, MD, USA). Agarose electrophoresis was used to evaluate the integrity of the total RNA samples. For each sample, a total of 2 µg of RNA was taken for RNA-seq library preparation. Briefly, mRNA was isolated from total RNA with the NEBNext Poly(A) mRNA Magnetic Isolation Module. The rRNA was removed from the total RNA with a RiboZero Magnetic Gold Kit. The enriched mRNA or rRNA-depleted RNA was subjected to the RNA-seq library preparation via a KAPA Stranded RNA-Seq Library Prep Kit (Roche). The library preparation procedure included fragmentation of the RNA molecules, reverse transcription to synthesize first-strand cDNA, second-strand cDNA synthesis incorporating dUTP, end repair and A-tailing of the double-stranded cDNA, Illumina compatible adapter ligation, and PCR amplification and purification of the final RNA-seq library. The completed libraries were analyzed on an Agilent 2100 Bioanalyzer for concentration, fragment size distribution between 400 and 600 bp, and adapter dimer contamination. The barcoded libraries were mixed in equal amounts and subjected to sequencing. The DNA fragments in the well-mixed libraries were denatured with 0.1 M NaOH to generate single-stranded DNA molecules, loaded onto the channels of the flow cell at an 8 pM concentration, and amplified in situ using a NovaSeq 6000 S4 Reagent Kit (300 cycles). Sequencing was carried out using an Illumina NovaSeq 6000 according to the manufacturer's instructions. Sequencing was carried out by running 150 cycles. The generated large RNA-seq data were analyzed to identify novel genes and pathways using differential gene expression analysis, pathway enrichment, and network analysis (Fig. 1D).

### Differential Expression Analysis

A total of 16 biological replicates from OFBI and naïve mouse corneas were subjected to differential gene expression (DGE) analysis with the aim of identifying genes that exhibited differential expression patterns in both samples. We used an established DGE analysis protocol from our previous studies^36^^–^^39^ via widely distributed Python and R software packages and bioinformatics tools.

#### Quality Check and Trimming

FastQC is a popular tool for evaluating the quality of unprocessed sequencing data. It provides a collection of analytics to quickly determine the quality of sequences based on summary graphs and tables pertaining to basic statistics, per-base sequence quality, sequence content, per-base N content, GC content, sequence length distribution, etc., FastQC (version 0.12.1).^40^ Cutadapt (version 4.6)^41^ was used to filter out adapter sequences, primers, poly-A tails, and other types of unwanted sequences from the high-throughput sequencing reads. We evaluated the quality of all 16 RNA sequences from OFBI (16 samples) and naïve (16 samples), and unwanted sequences were removed to ensure that high-quality reads were not filtered during the quality check.

#### Read Alignment

HISAT2^42^ was used for reads alignment of trimmed RNA sequences of both the OFBI and naïve samples with the mouse reference genome GRCm38. HISAT2 uses a novel genome indexing scheme by implementing a graph-based approach to capture a wide representation of genetic variant reads. Unlike other graph aligner methods (vg19 and bpa aligner20)^43^^,^^44^ that use memory-intensive k-mer-based indices, HISAT2 implements the graph FM (GFM) index. HISAT2 starts by constructing a linear graph of the reference genome and subsequently inserts, deletes, and identifies mutations as alternate pathways across the graph, thereby covering a wide representation of genetic variant reads. This approach makes HISAT2 an efficient and practical approach for the alignment of raw sequencing reads to a graph that records the entire mouse genome as well as a large number of variants.

#### Transcript Assembly

StringTie (version 2.2.1)^45^^,^^46^ was used for assembling mapped reads into transcripts via a network flow algorithm (initially established in optimization theory) and optional de novo assembly. Compared with other prominent transcript assembly tools, such as Cufflinks,^47^ IsoLasso,^48^ Scripture,^49^ and Traph,^50^ StringTie creates more comprehensive and accurate gene reconstructions and precise estimations of expression levels. Unlike Cufflinks, which finds a minimal set of transcripts (using a parsimony-based algorithm) and then estimates their expression levels separately, StringTie assembles transcripts and estimates their expression levels concurrently. The genome-guided transcript assemblers cluster the reads and generate graph models representing all potential isoforms for each gene using reads mapping to the reference genome. StringTie repeatedly selects the heaviest route from a splice graph, builds a flow network, calculates the maximum flow to estimate abundance, and then modifies the splice graph by eliminating reads assigned by the flow method. This method was repeated until all the reads were allocated, and a distinct flow network was constructed for each transcript to estimate its expression level using the maximum flow technique. Furthermore, the StringTie merge tool was used to construct a nonrepetitive set of transcripts identified in all the previously assembled RNA-seq datasets. StringTie merge was run with all assembled transcript files that were obtained for each sample, as well as a reference annotation file (gencode.vM25.annotation.gtf), and merged/assembled, global, and unified sets of transcripts (isoforms) from various RNA-seq samples were obtained as a single file.

#### Differentially Expressed Features

The gene count files obtained using StringTie for each sequence profile were used as input for DESeq2 (version 1.34.0)^51^ to identify the differentially expressed features. Annotate DESeq2/DEXSeq was used to add annotation information from the GTF file to the differentially expressed genes (DEGs). The aforementioned utility enhances the DESeq2/edgeR/limma/DEXSeq output table by including gene symbols, biotypes, and gene locations. The inclusion of data may be adjusted, and this information ought to be included in the input GTF/GFF file as attributes of the selected feature.

### Pathway Enrichment Analysis

Pathway enrichment analysis of DEGs was conducted using Gene Ontology (GO), Reactome, and Kyoto Encyclopedia of Genes and Genomes (KEGG) databases to identify critical pathways affected by OFBI. Hierarchical relationships among enriched terms and pathways were visualized through tree plots, whereas dot plots provided a quantitative overview of the most significantly enriched terms based on gene ratios and *P* values. This approach enabled the identification of key biological attributes and pathway-level insights with statistical significance set at *P* < 0.05, ensuring a robust and comprehensive understanding of the functional roles of the DEGs.

### Protein‒Protein Interaction Network Analysis

The STRING database (https://string-db.org) was used to construct and analyze protein‒protein interaction (PPI) networks by incorporating both experimentally validated and predicted interactions.^52^ These networks provide a comprehensive view of the direct physical associations and indirect functional relationships among mouse corneal proteins affected by the OFBI. PPI networks were derived from genes/proteins associated with the most significantly enriched pathways, facilitating the identification of molecular targets and key regulators affected by OFBI in mouse corneas. The hub genes were identified through network topology analysis using the PPI network. Cytoscape (version 3.8.2), coupled with the cytoHubba plugin, was used to detect potential targets and influential nodes within complex interaction networks.^53^^–^^55^ This analysis prioritized key hub genes that are likely to play pivotal roles in corneal OFBI and may offer insights into their functional significance and potential as therapeutic targets.

## Results

### RNA Sequence Quality Check

A quality assessment of RNA sequences from OFBI and naïve samples demonstrated excellent sequence quality, ensuring reliability for downstream analyses. A Phred quality score (Q) > 30, indicating a base call accuracy of 99.9%, was used as the benchmark. For both OFBI and naïve samples, more than 92% of the bases achieved this threshold, which reflects a very low probability of base call errors (Supplementary Table S1).

### Sequence Reads Alignment

After quality checks and preprocessing, RNA sequences from OFBI and naïve samples were aligned to the mouse reference genome (e.g. GRCm38) using HISAT2. The sequencing and mapping summary of the OFBI and naïve samples (Table 1) demonstrated high-quality data and efficient alignment to the reference genome. The initial raw read pairs ranged from approximately 15.4 million to approximately 19.9 million across samples, with a minimal reduction in trimmed reads after adaptor removal and filtering of reads shorter than 20 bp. The trimmed reads contained low proportions of mitochondrial RNAs (mtRNAs; = 0.89%–2.25%) and ribosomal RNAs (rRNAs; = 0.21%–0.56%), which indicate high-quality RNA extraction and preparation. Most trimmed reads were successfully aligned to the reference genome, with mapping rates exceeding 95% across all samples. The low proportion of unmapped reads (2.46%–4.45%) further reflects the robustness of the experimental setup, ensuring accurate differential gene expression and pathway enrichment analyses.

**Table 1.** Summary of Sequencing Read Mapping for the OFBI and Naïve Groups

Samples	Raw Pairs	Trimmed	mtRNAs	rRNAs	Mapped	Unmapped
OFBI.M1	17694423	17327354	1.57%	0.49%	96.79%	3.21%
OFBI.M2	16341135	15983370	1.25%	0.36%	97.28%	2.72%
OFBI.M3	15919692	15675240	1.65%	0.37%	97.30%	2.70%
OFBI.M4	15424322	15073310	1.29%	0.21%	97.36%	2.64%
OFBI.F1	18129208	17188934	2.25%	0.56%	95.55%	4.45%
OFBI.F2	16582845	16303743	1.41%	0.38%	97.07%	2.93%
OFBI.F3	18563246	17704489	1.40%	0.38%	97.02%	2.98%
OFBI.F4	19942887	19181720	1.26%	0.48%	96.87%	3.13%
Naïve.M1	16162060	15911840	1.63%	0.37%	97.47%	2.53%
Naïve.M2	16709006	16436721	1.53%	0.29%	97.10%	2.90%
Naïve.M3	16551554	16218853	1.05%	0.23%	96.39%	3.61%
Naïve.M4	17940198	17650692	0.89%	0.27%	96.35%	3.65%
Naïve.F1	19716698	19422473	1.59%	0.30%	97.20%	2.80%
Naïve.F2	19367297	18998701	1.68%	0.22%	97.54%	2.46%
Naïve.F3	16175140	15985148	1.30%	0.28%	97.39%	2.61%
Naïve.F4	19496063	19222512	0.90%	0.24%	96.88%	3.12%

The column “Raw Pairs” represents the initial number of sequencing fragments (read pairs), whereas “Trimmed” indicates the number of fragments remaining after 5′ and 3′ adaptor removal and filtering of reads shorter than 20 bp. The “Mapped” column represents the percentage of reads successfully aligned to the reference genome, whereas the “Unmapped” column indicates the percentage of reads that failed to align. In addition, the mitochondrial RNAs (mtRNAs) and ribosomal RNAs (rRNAs) within the trimmed sequences are shown.

### Differential Gene Expression Analysis

The resulting eight BAM files from the OFBI (8 cohorts) and naïve (8 cohorts) groups were processed using StringTie to assemble and quantify the transcripts. The resulting gene count files containing 15,948 annotations were submitted to DESeq2 for DGE analysis between the OFBI and naïve groups. A total of 2860 significantly expressed genes were identified with an adjusted *P* value < 0.05 (Supplementary Table S2). By applying cutoff values of log2 fold change ≥ 1.5 for upregulated genes and log2 fold change ≤ −1.5 for downregulated genes, 30 upregulated and 735 downregulated genes were identified. The top 10 up- and downregulated genes are given in Table 2.

**Table 2.** Top 10 Downregulated and Upregulated Differentially Expressed Genes in OFBI Mice Compared to Naïve Controls

DGE	Gene ID	Name	Log2 Fold Change	Adjusted *P* Value
Downregulated	*1500009C09Rik*	Known as Smim45-small integral membrane protein 45	–7.118	1.580E-13
	*Kcnh7*	Potassium voltage-gated channel subfamily H member 7	–6.112	6.830E-06
	*Srd5a2*	Steroid 5 alpha-reductase 2	–5.895	3.570E-05
	*Grik2*	Glutamate ionotropic receptor kainate type subunit 2	–5.675	2.830E-06
	*Glra2*	Glycine receptor alpha 2 subunit	–5.573	1.990E-06
	*Jakmip3*	Janus kinase and microtubule interacting protein 3	–5.467	5.855E-03
	*Crtac1*	Cartilage acidic protein 1	–5.406	6.020E-07
	*Smim17*	Small integral membrane protein 17	–5.317	1.280E-03
	*Pou4f2*	POU domain, class 4, transcription factor 2	–5.315	6.700E-04
	*Spry3*	Sprouty homolog 3	–5.138	7.560E-05
Upregulated	*Afm*	Afamin	7.648	2.540E-18
	*Caps2*	Calcyphosine 2	4.922	8.380E-10
	*Duxbl3*	Double homeobox B-like 3	4.030	3.218E-02
	*Duxbl2*	Double homeobox B-like 3	4.008	3.340E-02
	*Adamts18*	ADAM metallopeptidase with thrombospondin type 1 motif 18	3.725	4.810E-18
	*Ttpal*	Alpha-tocopherol transfer protein-like	3.235	2.640E-07
	*Cd96*	Cluster of differentiation 96	3.099	2.968E-02
	*Mid1*	Midline 1	2.336	3.390E-03
	*Cnpy1*	Canopy FGF signaling regulator 1	2.053	4.970E-18
	*Gbp8*	Guanylate-binding protein 8	1.933	2.040E-07

**Figure 2.**
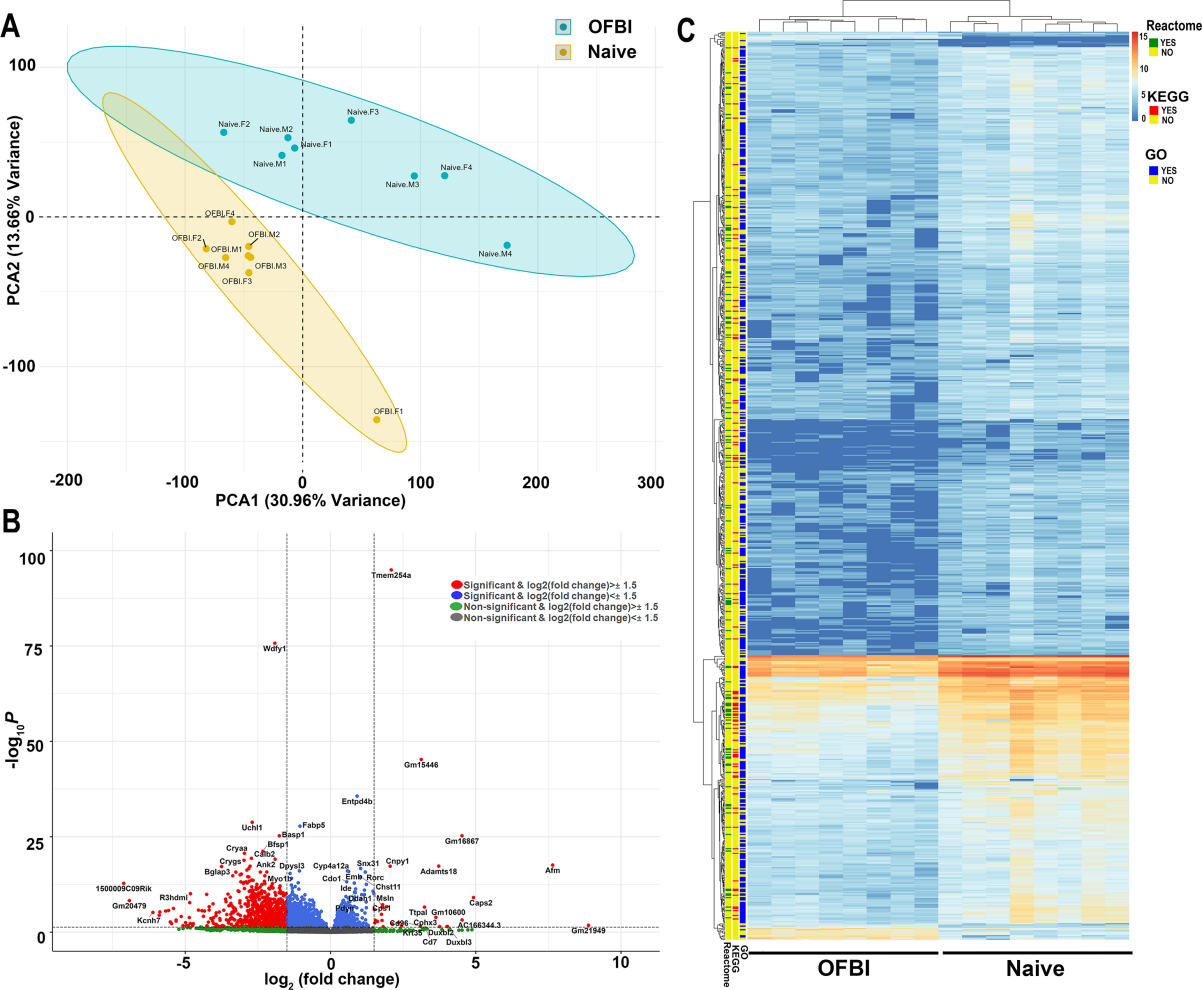
Distinct gene expression profiles between OFBI and naïve mouse corneas. (**A**) PCA plot showing clear separation between the OFBI (*orange*) and naïve (*blue*) groups based on gene expression profiles. The first two principal components (PCA1 and PCA2) explain 30.96% and 13.66% of the total variance, respectively. Each dot represents a sample, and ellipses indicate 95% confidence intervals for each group. (**B**) Volcano plot showing genes significantly up- and downregulated (*red*) in OFBI samples relative to naïve samples are shown based on their log2 fold change values on the x-axis and –log10 (*P* value) values on the y-axis. The *gray* and *blue points* represent nonsignificant genes beyond the log2 fold change threshold of ±1.5. (**C**) Heatmap showing hierarchical clustering of differentially expressed genes across OFBI and naïve samples. The gene expression values were z score normalized. Pathway annotations from Reactome, KEGG, and GO are shown on the *left* as colored bars, where “YES” indicates a significant pathway association and “NO” indicates otherwise.

The transcriptomic analysis revealed distinct differences between the OFBI and naïve groups, as visualized through multiple analytical approaches. Principal component analysis (PCA; Fig. 2A; based on gene counts) demonstrated clear segregation between the two groups, with PCA1 and PCA2 explaining 30.96% and 13.66% of the variance, respectively, which suggests significant transcriptomic alterations due to OFBI. The volcano plot (Fig. 2B) revealed DEGs, with significant upregulation and downregulation observed for key genes (log2 fold change > ±1.5 and adjusted *P* value < 0.05), indicating significant biological changes in response to the injury. The heatmap (Fig. 2C) displayed hierarchical clustering of DEGs, with distinct expression patterns separating the OFBI and naïve groups. Enrichment analyses highlighted associations with pathways annotated using Reactome, KEGG, and GO databases.

### Pathway Enrichment Analysis

**Figure 3.**
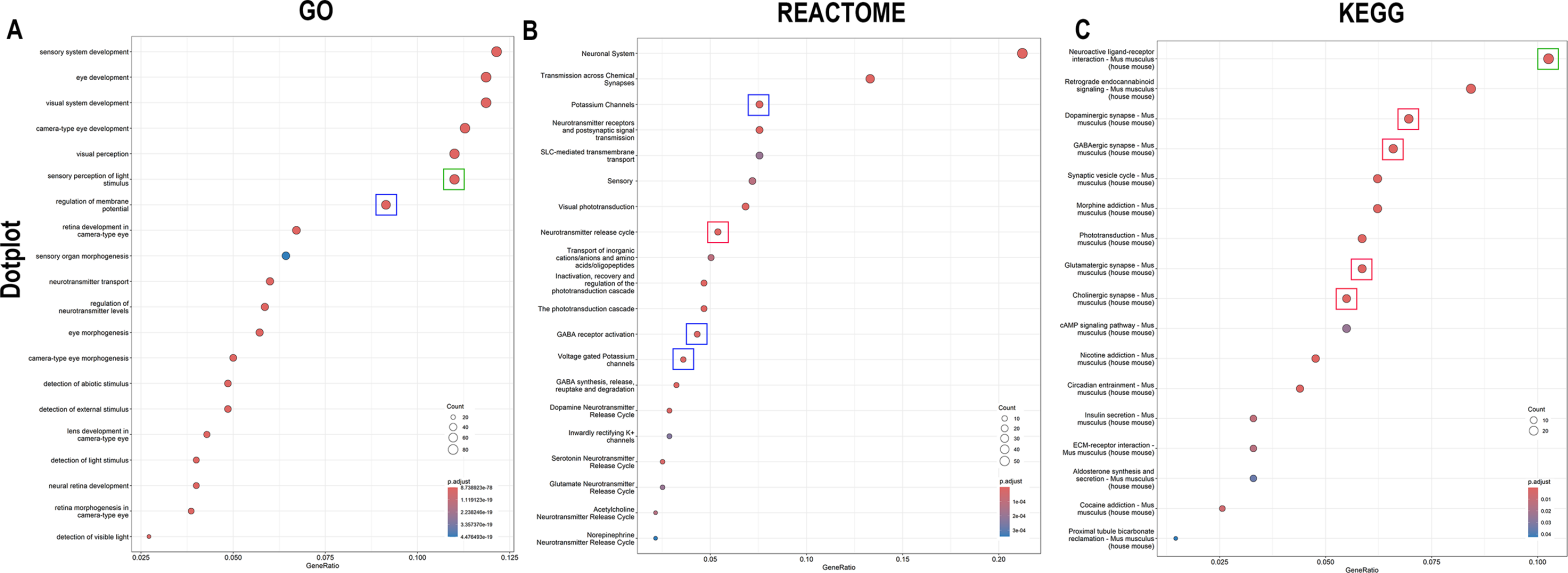
Pathway enrichment analysis of DEGs for day 14 post-OFBI using Gene Ontology (GO), Reactome, and KEGG databases. Figure 2A to 2C are dot plots showing the most significantly enriched terms for the GO (**A**), Reactome (**B**), and KEGG (**C**) analyses. The *blue*, *green*, and *red rectangles* represent the selected pathways for ion channels and membrane potential, sensory perception, and neurotransmitter signaling, respectively. The size of each dot corresponds to the number of genes involved in the pathway, whereas the color intensity indicates statistical significance (adjusted *P* value < 0.05).

The results of the pathway enrichment analysis of the DEGs via the GO, Reactome, and KEGG databases are represented in both hierarchical tree plots and dot plots, which provide a detailed understanding of the functional attributes and pathway-level insights (Fig. 3). Among the enriched pathways identified, we focused on those most relevant to corneal function, specifically pathways influencing ion channels and membrane potential, sensory perception, and neurotransmitter transport and regulation. This allowed us to narrow our study and focus on these critical functionalities.

**Figure 4.**
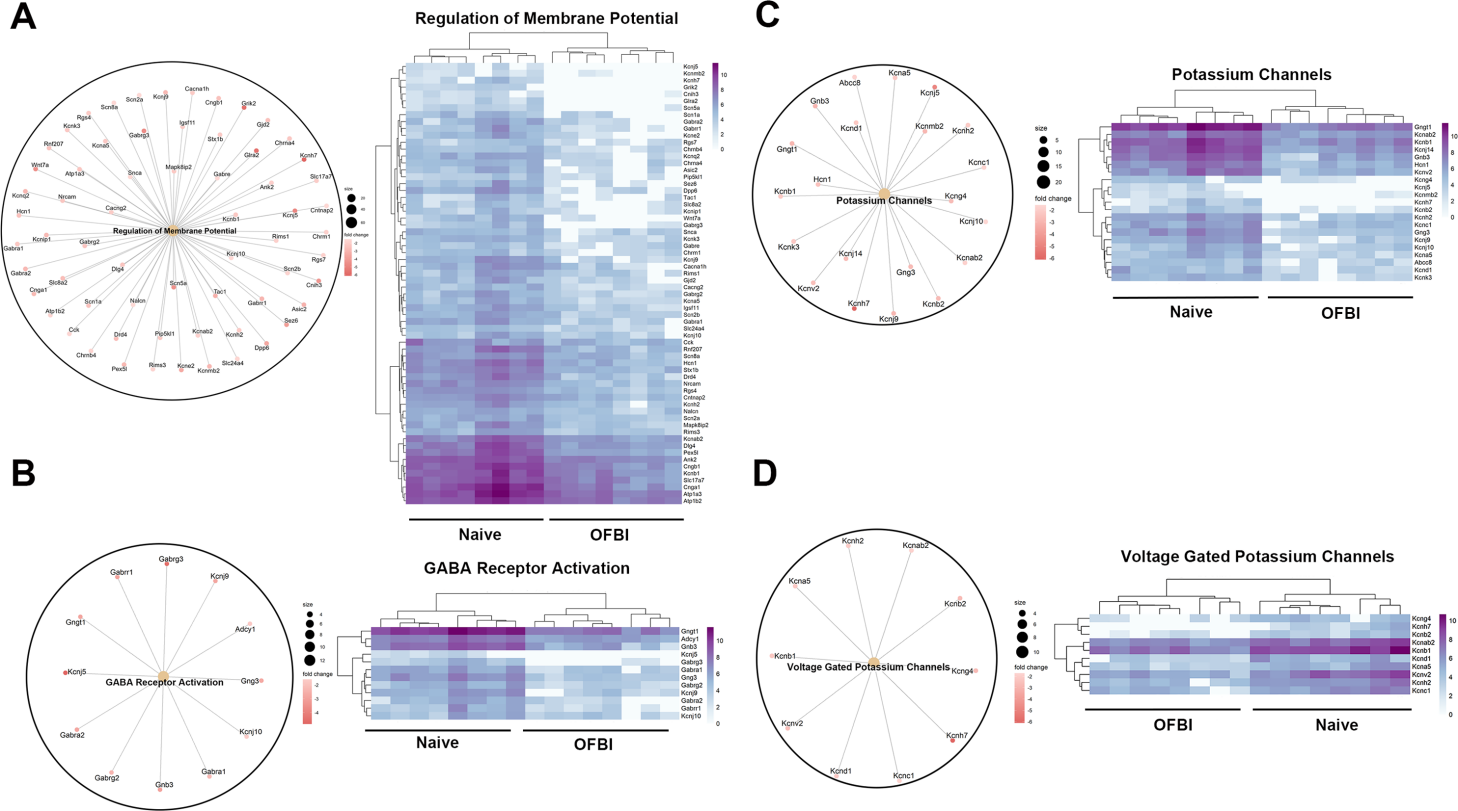
Gene clusters and corresponding heatmaps of enriched pathways related to corneal ion channels and membrane potential following OFBI on day 14. (**A**) Regulation of the membrane potential. (**B**) GABA receptor activation. (**C**) Potassium channels. (**D**) Voltage-gated potassium channels. In each panel, the *left circle plot* shows the genes contributing to the pathway, with the size of the dots representing gene significance (adjusted *P* value < 0.05). The right heatmap shows the differential expression of these genes between the naïve and OFBI conditions. The upregulated and downregulated genes are represented by intensity variations in the heatmap, with *purple* indicating higher expression and lighter shades indicating lower expression.

**Figure 5.**
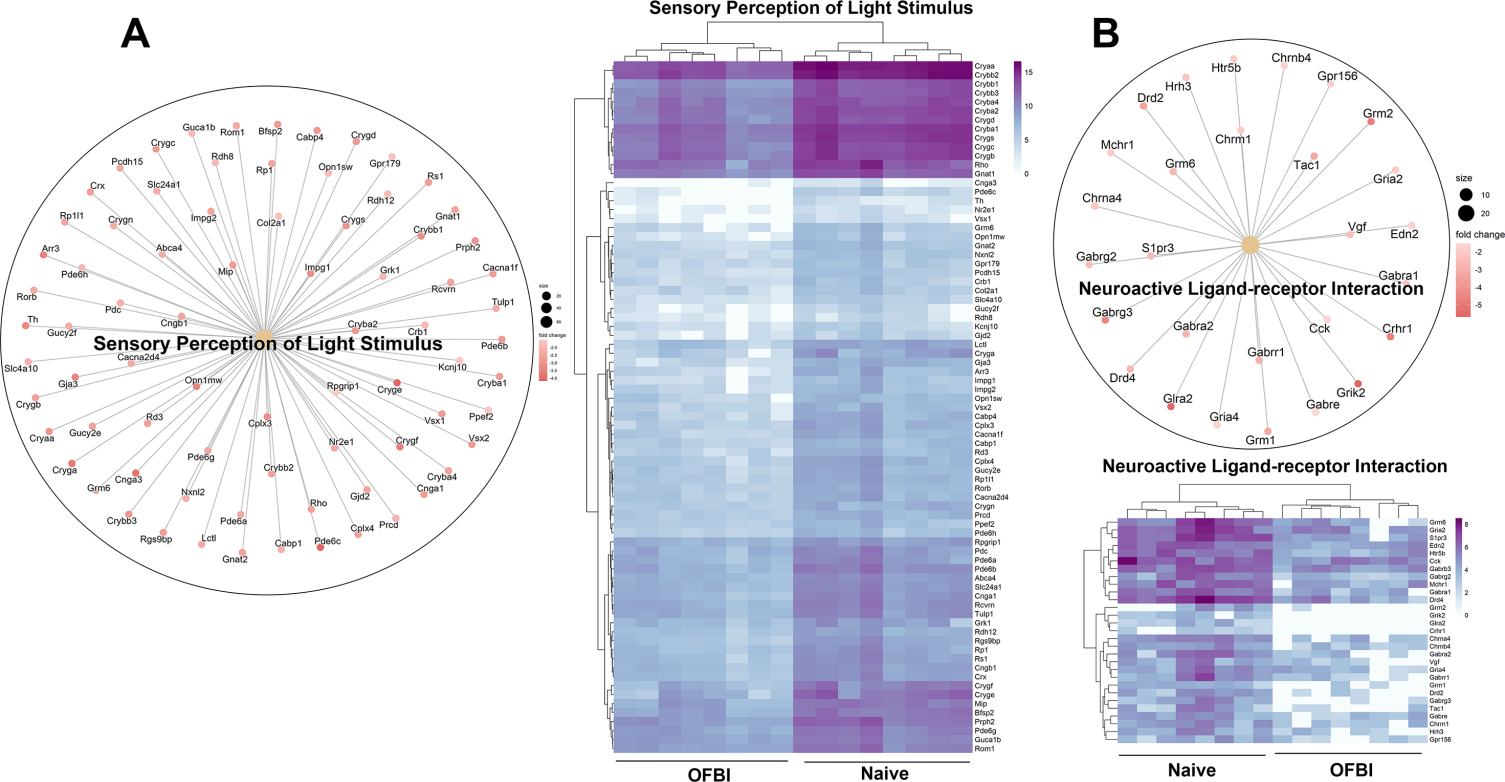
Enriched pathways associated with corneal sensory perception on day 14 following OFBI. (**A**) Sensory perception of the light stimulus. (**B**) Neuroactive ligand‒receptor interaction. The *left circular plot* illustrates genes involved in detecting light stimuli, with the sizes of the dots representing the statistical significance of each gene (adjusted *P* value < 0.05). The right heatmap shows the differential expression of these genes between the naïve and OFBI conditions, with *purple* indicating upregulation and lighter shading indicating downregulation.

**Figure 6.**
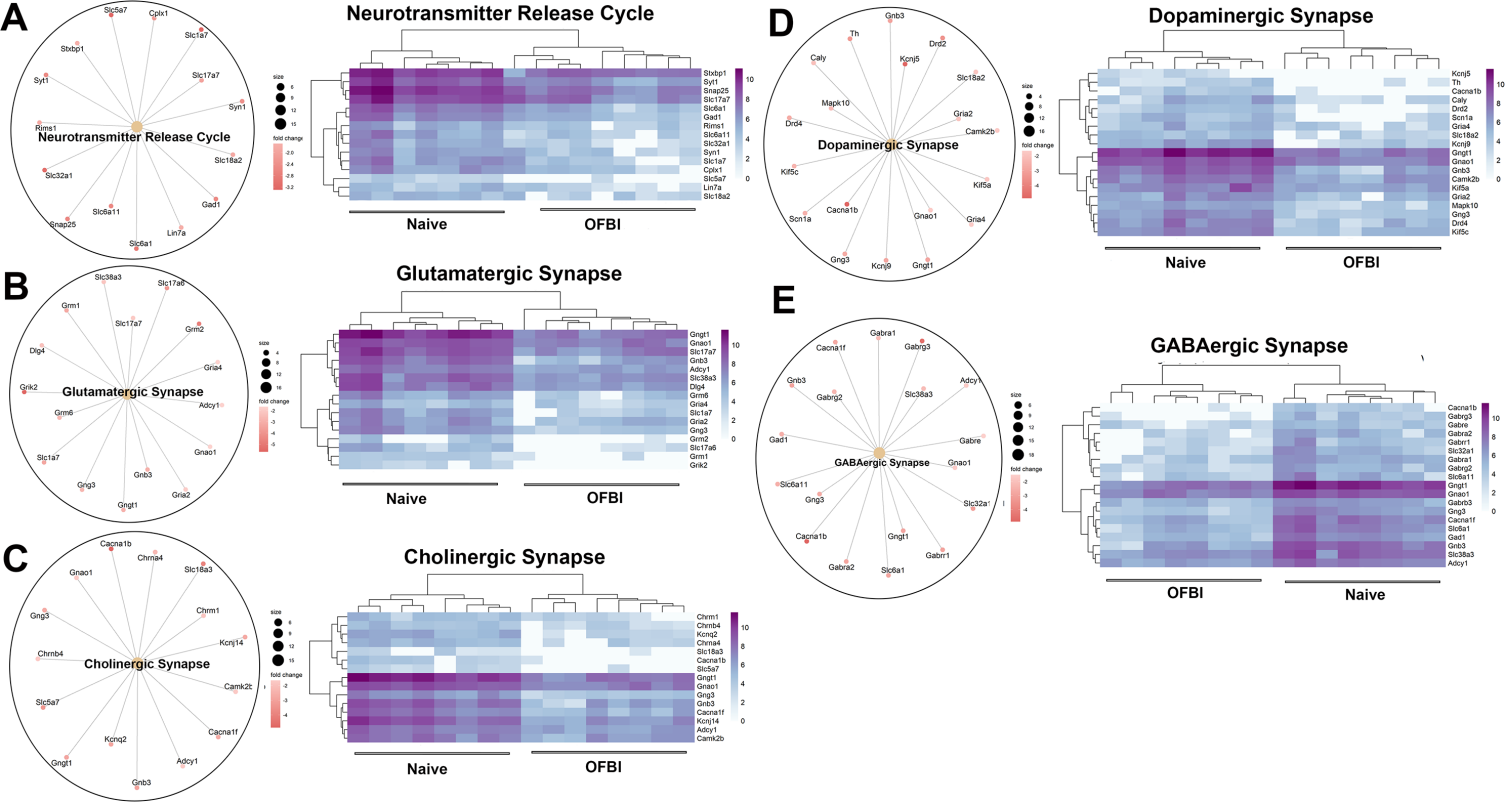
Gene clusters and corresponding heatmaps of pathways related to neurotransmitter signaling related to corneal function following OFBI. (**A**) Neurotransmitter release cycle. (**B**) Glutamatergic synapse. (**C**) Cholinergic synapse. (**D**) Dopaminergic synapse. (**E**) GABAergic synapse. For each pathway, the *left circular plot* shows genes contributing to the pathway, with the dot size indicating the statistical significance of each gene (adjusted *P* value < 0.05). The right heatmap displays the differential gene expression levels between the naïve and OFBI conditions, with *purple shades* representing upregulation and lighter shades indicating downregulation.

Ion channel and membrane potential regulation includes pathways, the regulation of membrane potential, GABA receptor activation, potassium channels, and voltage-gated potassium channels (Fig. 4). The enriched pathways associated with sensory perception were the sensory perception of light stimulus pathway and the neuroactive ligand‒receptor interaction pathway (Fig. 5). The neurotransmitter transport and regulation pathways included the neurotransmitter release cycle, glutamatergic synapse, cholinergic synapse, dopaminergic synapse, and GABAergic synapse (Fig. 6).

### Protein‒Protein Interaction Network Analysis

**Figure 7.**
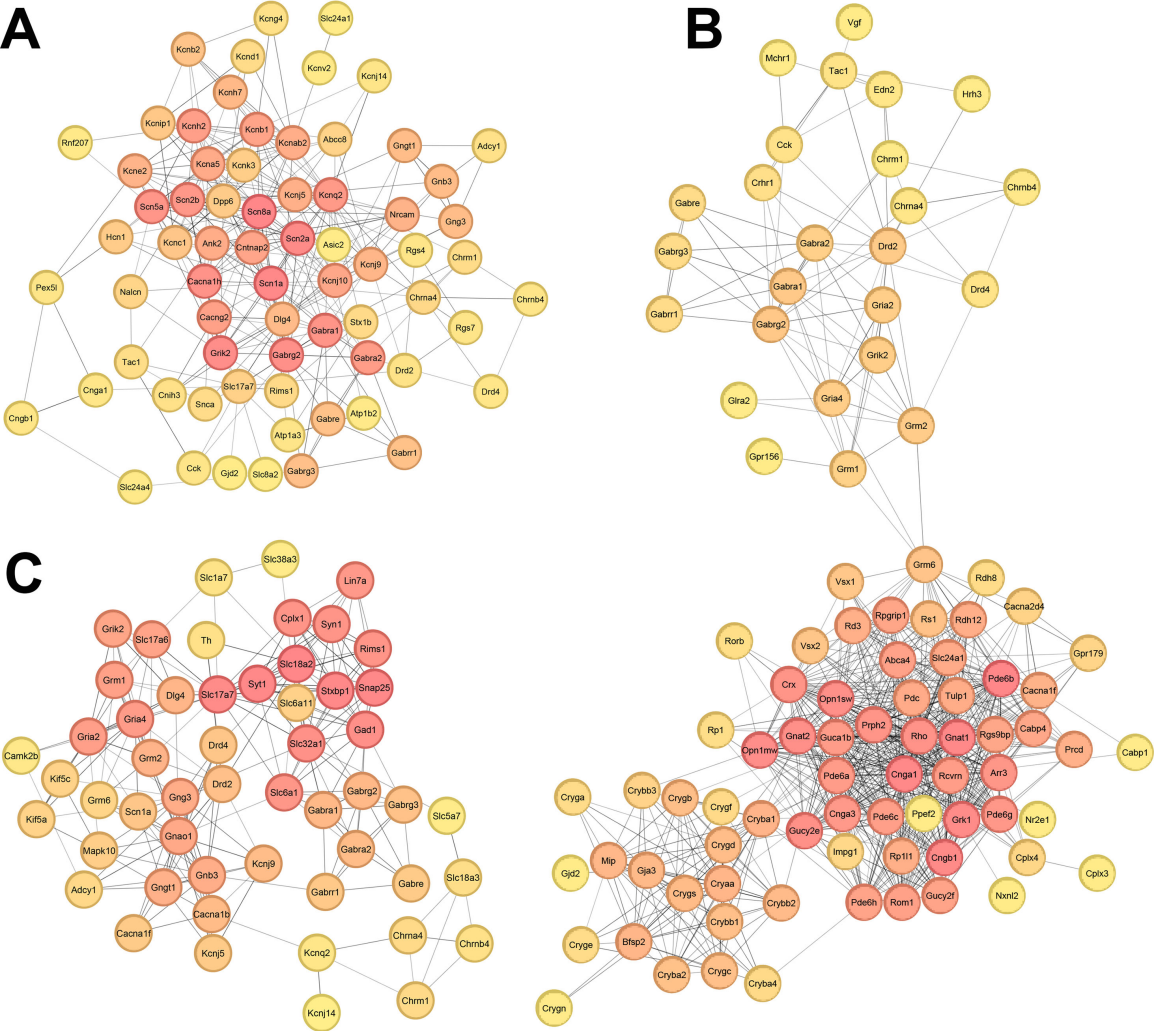
Protein‒protein interaction networks of pathways influencing (**A**) ion channels and membrane potential, (**B**) sensory perception, and (**C**) neurotransmitter transport and regulation on day 14 post-OFBI. Each network represents the union of relevant pathways by common nodes for its respective category generated using Cytoscape. Node ranking was performed via the CytoHubba module, with node color intensity reflecting the ranking: *deep red* indicates stronger involvement, and *yellow* indicates weaker involvement in the network.

PPI network analysis revealed key insights into the molecular pathways disrupted in the cornea following OFBI. Using the STRING database and Cytoscape, three distinct networks were generated to represent pathways associated with ion channels and membrane potential (Fig. 7A), sensory perception (Fig. 7B), and neurotransmitter transport and regulation (Fig. 7C). Each network highlights direct physical interactions and indirect functional relationships among corneal proteins affected by OFBI.

The ion channels and membrane potential network (see Fig. 7A) integrate pathways, including the regulation of the membrane potential, GABA receptor activation, potassium channels, and voltage-gated potassium channels. The sensory perception network (see Fig. 7B) integrates pathways, such as sensory perception of light stimulus and neuroactive ligand–receptor interaction. The neurotransmitter transport and regulation network (see Fig. 7C) encompasses pathways including the neurotransmitter release cycle, glutamatergic synapse, cholinergic synapse, dopaminergic synapse, and GABAergic synapse.

## Discussion

The comprehensive transcriptomic analysis presented here provides significant insights into the molecular disruptions in the cornea on day 14 in vivo post-open-field real-time blast/explosive detonations in which changes in corneal transcriptomics are cumulative net effect of internal (intraocular tissue damage) and external (shockwaves, debris, dust particles, etc.) factors. High-quality sequencing (see Supplementary Table S1) and alignment metrics (see Table 1) highlight the reliability of the results. Subsequent DGE (see Supplementary Table S2) and pathway analyses revealed critical pathways affected by OFBI, particularly those related to ion channels and membrane potential, sensory perception, and neurotransmitter regulation (see Fig. 3).

The distinction between OFBI and naïve samples observed via PCA and heatmaps emphasizes significant transcriptomic alterations in response to OFBI on day 14 and validates the biological impact of this injury (see Fig. 2). The identification of 30 upregulated and 735 downregulated genes (log2 fold change ≥ ±1.5) revealed extensive transcriptomic changes, with notable impacts on ion channels, neurotransmitter signaling, and sensory pathways. The substantial downregulation of genes such as *Kcnh7*, *Grik2*, and *Glra2*, which are integral to maintaining ionic balance and excitatory/inhibitory signaling in nonocular tissues,^56^^,^^57^ may lead to disrupted homeostasis in corneal nerves on day 14 post-OFBI (see Table 2). The identified DEGs with a large number of downregulated genes (735) compared with a smaller number of upregulated genes (30) provide a foundation for understanding how OFBI alters corneal neuronal and structural integrity.

Among all the enriched pathways, those involved in ion channels and membrane potential, sensory perception, and neurotransmitter transport and regulation were selected for further analysis of OFBI corneas. These three functional categories were selected based on their critical roles in corneal physiology and their potential disruption following traumatic injury. Ion channels are essential for regulating ionic gradients across corneal endothelial and epithelial cells.^58^^,^^59^ This regulation is necessary for fluid transport and maintaining corneal transparency, and dysfunction in these channels can lead to corneal edema, impairing vision. The enrichment of pathways, such as those related to the regulation of membrane potential, GABA receptor activation, and potassium channels highlights the disruption of ionic homeostasis and neuronal excitability in the cornea (see Fig. 4). The downregulation of ion channel-related genes, such as *Kcnj5*, *Scn5a*, and *Kcnq2* in OFBI corneas, correlates with impaired ionic homeostasis and nerve hyperexcitability. Potassium channels, which are essential for maintaining the resting membrane potential and regulating action potentials, are particularly vulnerable.^60^ The dysregulation of these pathways could result in altered sensory nerve signaling, leading to corneal hypoesthesia or neuropathic pain.

Pathways such as sensory perception of light stimulus and neuroactive ligand-receptor interaction highlight potential impairments in the corneal sensory transduction process (see Fig. 5). These pathways seem critical for maintaining corneal sensitivity and reflexive tear production.^61^ The disruption of these pathways could affect the corneal reflex and sensory signaling and may lead to damage or degeneration of corneal sensory nerves, resulting in hypoesthesia, disruption of corneal homeostasis, and exacerbation of corneal inflammation and dry eye symptoms, which are commonly observed in blast-injured eyes.^62^^,^^63^

The enrichment of pathways including glutamatergic synapse, cholinergic synapse, and GABAergic synapse reflects significant alterations in neurotransmitter dynamics within the cornea (see Fig. 6).^64^^–^^67^ The downregulation of critical genes, such as *Grik2*, *Gabra2*, *Gabrr1*, *Gabra1*, *Syt1*, and *Slc32a1*, suggests impaired synaptic transmission and neurotransmitter release.^68^ Although these genes are not extensively characterized in the cornea, their disruption is likely to affect the excitatory and inhibitory signaling balance in the cornea, thereby altering sensory processing.

The PPI network analyses highlighted central nodes and their interconnectivity within critical pathways (see Fig. 7). Through network analyses, 12 nodes were found to be common to all 3 functional categories, that is, ion channels and membrane potential, sensory perception, and neurotransmitter transport and regulation (Table 3). Among these common nodes, glutamate ionotropic receptor kainate type subunit 2’ (*Grik2*/*Gluk2*) was most downregulated (log2 fold change = –5.675) in OFBI corneas compared with naïve corneas. Studies have shown that glutamate, along with other neurotransmitters, is present in the cornea; however, its precise role in corneal physiology remains largely unclear.^69^^,^^70^ *Grik2*, which encodes kainate receptor subunit 2 from the kainate receptor family, plays a key role in mediating excitatory neurotransmission by binding to kainate (a subtype of glutamate). This receptor regulates excitatory signaling and slower synaptic events. In the cornea, significant downregulation of *Grik2* (approximately 48-fold greater in OFBI corneas than in naïve corneas) appears to be among the major events associated with potential dysregulation of excitatory signaling in the cornea.

**Table 3.** Lists of the 12 Protein Nodes That are Common to the Three Considered Functional Categories: Ion Channels and Membrane Potential, Sensory Perception, and Neurotransmitter Transport and Regulation

Protein ID	Name	Gene log2 Fold Change	Adj *P* Value
Grik2	Glutamate ionotropic receptor kainate type subunit 2	–5.675	2.830E-06
Gabrg3	Gamma-aminobutyric acid type A receptor subunit gamma 3	–4.544	1.630E-06
Drd2	Dopamine receptor D2	–3.439	2.297E-04
Gabrr1	Gamma-aminobutyric acid type C receptor subunit rho 1	–2.941	3.422E-04
Gabra2	Gamma-aminobutyric acid type A receptor subunit alpha 2	–2.833	1.093E-03
Drd4	Dopamine receptor D4	–2.674	4.210E-07
Gabrg2	Gamma-aminobutyric acid type A receptor subunit gamma 2	–2.327	1.940E-05
Chrna4	Cholinergic receptor nicotinic alpha 4 subunit	–2.213	3.047E-03
Gabra1	Gamma-aminobutyric acid type A receptor subunit alpha 1	–2.199	1.693E-04
Chrm1	Cholinergic receptor muscarinic 1	–1.864	1.108E-02
Chrnb4	Cholinergic receptor nicotinic beta 4 subunit	–1.692	3.684E-03
Gabre	Gamma-aminobutyric acid type A receptor subunit epsilon	–1.515	2.351E-02

The transcriptomic alterations observed post-OFBI align with known pathological features, such as corneal nerve damage, impaired wound healing, and sensory dysfunction. The identified pathways may provide potential therapeutic targets for mitigating OFBI-induced corneal damage. For example, modulating potassium channels or GABAergic signaling could restore ionic balance and nerve function, targeting pathways involved in sensory perception may improve corneal sensitivity and reflexes, and enhancing neurotransmitter regulation may alleviate neuropathic pain and support neuronal repair.

This study provides a detailed transcriptomic framework for OFBI-induced corneal changes; however, certain limitations exist. The RNA-seq of corneal tissue provides a high-throughput overview of net transcriptional alterations; however, it does not provide information for individual corneal cells, epithelium, stroma, or endothelium. To address this potential limitation, future studies should use single-cell RNA sequencing or spatial transcriptomics to dissect cell-type-specific responses and further investigate regional variations within corneal cells and adjacent ocular tissues. In addition, the transcriptomic data in this study was captured at a single timepoint (day 14), the observed gene expression patterns may reflect a dynamic interplay between ongoing injury responses and overlapping repair processes. As such, pathway-level interpretations should be viewed as correlative rather than definitive. Thus, exploring the temporal dynamics of gene expression before and after day 14 would offer additional insights into the progression of corneal recovery or degeneration post-OFBI. Another perceived limitation is the lack of validation of transcriptomics data through proteomic and functional studies to confirm the biological relevance of identified pathways. Although the identification of ion channel disruption and neurotransmitter pathway alterations provides mechanistic clues, functional validation through protein-level assays, pharmacological inhibition, loss-of-function, and gain-of-function transgenic models are needed to confirm the roles of identified specific genes and networks in OFBI-induced corneal pathobiology. These are active areas of investigation in our laboratory.

The utilized OFBI setup enabled simultaneous monitoring of blast wave dynamics, including peak overpressure and impulse, from multiple orientations. This research design ensured consistent subject positioning and accurate, reproducible pressure characterization during blast events. The transcriptomic analyses for male and female cohorts of OFBI groups individually showed no notable differences (Supplementary Table S3). It appears that the OFBI has no sex-dependent corneal morbidities, however, additional studies are warranted.

In conclusion, this study identifies the significant transcriptomics disruptions in ion channels, sensory perception, and neurotransmitter signaling in the cornea following OFBI. In addition, these findings not only provide the blueprint of the OFBI-induced mRNA changes in the cornea but also lay the foundation for future studies to develop therapies for corneal blindness resulting from explosive detonations.

## Supplementary Material

Supplement 1

Supplement 2

Supplement 3
