# Supplementary material for: Open-Field Blast Injury Disrupts Corneal Gene Expression Linked to Ion Transport, Sensory Perception, and Neural Signaling
Source: Invest Ophthalmol Vis Sci. 2025 Aug 27;66(11):68. doi: 10.1167/iovs.66.11.68 (PMC12395823; doi:10.1167/iovs.66.11.68)
Supplement: Supplement 1 [file iovs-66-11-68_s001.docx]

**Table S1**: Quality score of the OFBI and naïve corneal sequences.

| **Sample Name** | **Reads Count** | **Bases Num** | **Bases Num (Q ≥ 30)** | **Q30 (%)** |
| --- | --- | --- | --- | --- |
| OFBI.M1 | 35388846 | 5308326900 | 4956022364 | 93.36% |
| OFBI.M2 | 32682270 | 4902340500 | 4577245622 | 93.37% |
| OFBI.M3 | 31839384 | 4775907600 | 4467461447 | 93.54% |
| OFBI.M4 | 30848644 | 4627296600 | 4315928347 | 93.27% |
| OFBI.F1 | 36258416 | 5438762400 | 5051551930 | 92.88% |
| OFBI.F2 | 33165690 | 4974853500 | 4649858505 | 93.47% |
| OFBI.F3 | 37126492 | 5568973800 | 5156460715 | 92.59% |
| OFBI.F4 | 39885774 | 5982866100 | 5551261284 | 92.79% |
| Naïve.M1 | 32324120 | 4848618000 | 4555595268 | 93.96% |
| Naïve.M2 | 33418012 | 5012701800 | 4681714949 | 93.40% |
| Naïve.M3 | 33103108 | 4965466200 | 4632708622 | 93.30% |
| Naïve.M4 | 35880396 | 5382059400 | 5031070302 | 93.48% |
| Naïve.F1 | 39433396 | 5915009400 | 5533668660 | 93.55% |
| Naïve.F2 | 38734594 | 5810189100 | 5427022833 | 93.41% |
| Naïve.F3 | 32350280 | 4852542000 | 4570187846 | 94.18% |
| Naïve.F4 | 38992126 | 5848818900 | 5481868297 | 93.73% |
